# Tolerance of Broilers to Dietary Supplementation with High Levels of the DHA-Rich Microalga, *Aurantiochytrium Limacinum*: Effects on Health and Productivity

**DOI:** 10.3390/ani8100180

**Published:** 2018-10-16

**Authors:** Colm A. Moran, Douglas Currie, Jason D. Keegan, Anne Knox

**Affiliations:** 1 Regulatory Affairs Department, Alltech SARL, Vire, Rue Charles Amand, 14500 Vire, France; 2 Roslin Nutrition Ltd., Gosford Estate, Aberlady EH32 0PX, UK; Douglas.Currie@roslinnutrition.co.uk (D.C.); Anne.Knox@roslinnutrition.co.uk (A.K.); 3 Regulatory Affairs Department, Alltech European Bioscience Centre, A86 X006 Meath, Ireland; jkeegan@alltech.com

**Keywords:** broilers, DHA, omega-3, fatty acids, tolerance, safety

## Abstract

**Simple Summary:**

Docosahexaenoic acid (DHA) is an omega-3 fatty acid that is considered an essential part of the human diet. Increasing the amount of DHA in commonly consumed foodstuffs can benefit people predisposed to heart problems, depression, and even some forms of cancer. One of the best sources of DHA is oily fish, but the majority of people do not eat fish regularly. By formulating diets for livestock or poultry to include ingredients rich in omega-3 fatty acids, we can increase the omega-3 content of their respective meat and tissues. In this study we fed broilers diets supplemented with increasing amounts of a DHA-rich microalgae to investigate whether it was safe for the birds, in terms of their health and productivity and effective in terms of DHA transfer from feed to meat. The results of the present study showed that feeding the microalgae to chickens had no negative effects on their health in terms of their level of survival or growth. Moreover, we found that supplementing the microalgae resulted in a large increase in the DHA content of meat. This study demonstrated that feeding algae is a safe and effective way to improve the nutritional value of chicken meat.

**Abstract:**

It is well established that the docosahexaenoic acid (DHA) content of commonly consumed meats, such as chicken, can be increased through dietary supplementation with DHA-rich ingredients. The purpose of this study was to investigate the tolerance of broilers to dietary supplementation with the unextracted biomass of a DHA-rich microalgae *Aurantiochytrium limacinum*, so as to ensure its safety, since it is accumulated in broiler meat. Healthy day-old male Ross 308 chicks (*n* = 1120) were evenly distributed to 32 pens (35 chicks per pen), with pens randomly allocated to one of four dietary treatments, each having eight replicates. The dietary groups included one untreated control and three treatments corresponding to three inclusion levels (0.5, 2.5, and 5.0%) of All-G-Rich^®^, with the birds receiving the experimental diets ad libitum during the study (day 0–42). Bird survival, blood parameters, productivity, and breast and thigh DHA content were determined after 42 days of feeding. Supplementation at up to 10 times the intended use level had no negative effects on the mortality, blood parameters or productivity of the birds, while significant increases in the meat DHA content were observed. These results indicate that supplementation with *Aurantiochytrium limacinum* is a safe and effective way to increase broiler tissue DHA content.

## 1. Introduction

The health benefits associated with an increased intake of long chain omega-3 fatty acids (n-3 FAs), particularly eicosapentaenoic acid (EPA) and docosahexaenoic acid (DHA), are becoming increasingly clear [1]. Long chain n-3 FAs have been shown to decrease the risk of some cardiovascular diseases [2], can be beneficial in the treatment of some types of cancer [3], can improve higher cognitive functions when provided to infants [4], and can reduce cognitive decline for the elderly [5]. However, in many parts of the world, these benefits are not feasible for the population as insufficient quantities of n-3 FAs are consumed through diet. Long chain n-3 FAs are found in the highest concentrations in oily fish, and as a result, n-3 FA intake is often highly associated with fish consumption [6]. However, in many western countries, only small proportions of the population regularly consume fish [7], with those not consuming fish relying on other sources of n-3 FA. In Australia, for example, meat and poultry products provide up to 45% of the long chain n-3 FAs consumed by adults [6]. As such, increasing the n-3 FA content of commonly consumed meats, such as chicken, is an attractive method for enhancing the n-3 FA consumption of the population [8].

The fatty acid composition of broiler tissues can be easily modified, with an increased dietary fatty acid content leading to an increased fatty acid content in poultry tissue [9]. Historically, this was achieved through feeding n-3 FA rich fish oils to broilers; however, this can result in a reduced shelf life and can have a negative impact on the organoleptic properties of the meat [9,10]. In addition, due to declining fish stocks, using fish products to supplement animal diets is not considered a sustainable method of enriching meat [11]. Microalgae, one of the primary producers of long chain n-3 FAs in the food chain, can be grown in a sustainable manner on simple carbon sources and fed to livestock to increase the n-3 FA content of their edible products [9,12]. The marine microalgae, *Schizochytrium* sp. has been shown to increase the DHA content of broiler meat [13], while maintaining consumer acceptability without reducing shelf life [14]. *Aurantiochytrium limacinum,* a related microalgae, when grown heterotrophically on a low-sodium medium has also been shown to increase the DHA content of cows’ milk [15,16], of pig muscle tissue [17,18], and hens’ eggs [19].

The aim of this study was to assess the safety of feeding high levels of an unextracted biomass of the DHA-rich microalgae, *Aurantiochytrium limacinum*, to broiler chickens by investigating the effects of supplementation on broiler productivity, mortality, blood biochemistry, and hematology.

## 2. Materials and Methods

The research protocol and animal care were conducted in accordance with European Union Directive 2010/63/EU pertaining to the protection of animals used for experimental or other scientific purposes. The live animal portion of the study was conducted at Roslin Nutrition Ltd. Aberlady, UK. Following the standard operating procedures of Roslin Nutrition, ethical approval was not required as the substance under investigation is a registered feed ingredient in the EU. The study investigated the tolerance of broilers to supplementation with high levels of the heterotrophically grown microalgae *A. limacinum* (AURA; ALL-G-RICH^®^; CCAP 4087/2; Alltech Inc., Nicholasville, KY, USA), when fed over a 42-day period. The study was designed in keeping with the European Food Safety Authority (EFSA) guidance on how to conduct and report studies concerning safety for the target animal (tolerance studies) [20]. The birds were kept 35 per pen in a house equipped with 32 pens. The building was supplied with artificial programmable lights, automated gas heating, and forced ventilation. Temperature inside the building was as recommended by the breeder. The lighting programme was 23 h light and 1 h dark during each 24-h period throughout the trial. Fresh wood shavings were provided to a depth of approximately 10 cm in each pen. Pens were constructed from timber, plywood and wire mesh and measured 2.04 m × 1.51 m.

Day-old, male Ross 308 chicks (*n* = 1120) were evenly distributed among 32 pens, with each pen randomly assigned to one of four treatments: 0%; 0.5%; 2.5%; or 5% AURA. The basal diet ingredients are shown in Table 1, and were designed to meet or exceed the nutrient levels recommended for the Ross 308 broilers [21]. Both starter (from day 0–21) and grower (from day 21–42) diets were supplied ad libitum as 3-mm pellets. The AURA supplement was added to the diets as a top dressing. The proximate analysis composition of the experimental diets was determined at DM Scientific (Dalton, UK), with crude protein by the Dumas procedure by means of a FP-528 nitrogen/protein determinator (LECO Corp. St. Joseph, MO, USA), crude fat (oil A) (AOAC 920.39), dry matter by oven drying (AOAC 934.01) and ash by incineration (AOAC 967.05). Samples of each diet were ground and mixed until homogenous and the fatty acid content was then determined at the Mylnefield Institute (James Hutton Ltd., Dundee, UK). In brief, the fatty acids in each sample were transesterified in situ with 1.5 N HCl in methanol, in the presence of toluene. The toluene contained methyl tricosanoate which acted as the internal standard. The resultant fatty acid methyl esters (FAMEs) and toluene were then extracted. The FAMEs were then separated, identified, and quantified by gas chromatography. The mg of FAME per 100 g of sample was then calculated using the following formula:

$$\mathrm{FAME},\;\mathrm{mg}\;\mathrm{FA}/100\;g\;\mathrm{wet}\;\mathrm{sample}=\frac{A_{X}\times CF_{X}\times W_{IS}\times S\times 1000\times 100}{A_{IS}\times W_{S}\times 1.04\times 100}$$

where *A_X_* = area counts for EPA, DHA or GLA; *A_IS_* = area counts for internal standard; *CF_X_* = theoretical correction factor relative to C23:0 (IS); EPA = 0.98, DHA = 0.97, GLA = 1.01; *W_IS_* = weight of IS added to sample in mg; *W_S_* = weight of sample in mg; *S* = % solids of initial sample.

Daily bird health, mortality, and culling records were maintained. Any bird that died for unknown reasons, with the exception of non-starters (birds under 7 days of age) underwent a post-mortem examination by a veterinarians from St. David’s Poultry Team, UK. On days 0, 21, and 42, animal body weight and the amount of feed provided were measured per pen with feed refusals recorded on days 21 and 42. On day 42, blood samples (3 mL) were taken from the vena basilica of the left wing of one bird per pen (8 replicates per treatment) for biochemistry (heparin tube) and hematology (EDTA tube) by veterinarians from St. David’s Poultry Team, UK, who are licensed by the Home Office, UK, to carry out such procedures. The heparinized blood was centrifuged at 4 °C for 10 min after which plasma was removed and samples were stored at −80 °C in Eppendorf tubes until analysis. Blood hematology was conducted on the day of sampling and the variables included: basophils; eosinophils; hemoglobin; heterophils; lymphocytes; mean corpuscular volume; monocytes; packed cell volume; red blood cell count; and white blood cell count. The blood biochemistry variables included: alkaline phosphatase; aspartate amino transferase; calcium; cholesterol; creatine; glucose; glutathione peroxidase; lactose dehydrogenase; magnesium phosphate; triglycerides; urea; and uric acid. All blood analyses were conducted at SRUC Laboratories (Edinburgh, UK). The fatty acid content of chicken breast and thigh samples was determined for two birds per pen at the Mylnefield Institute (James Hutton Ltd., Dundee, UK). In brief, samples of chicken (breast and thigh) tissues were ground thoroughly using a mortar and pestle and left in a freezer at −16 °C for 12 h prior to freeze drying. The fatty acids in each sample were then transesterified in situ with 1.5 N HCl in methanol, in the presence of toluene. Methyl tricosanoate was present in the toluene and acted as the internal standard. The FAMEs and toluene were the extracted, separated, identified, and quantified using gas chromatography as described previously.

Differences between the treatment groups were determined using the general linear model procedure of Minitab^®^ (Minitab, v18, State College, PA, USA) with Tukey’s post hoc analysis used to determine the differences between the treatment groups. Regression analysis was used to determine whether the estimated DHA intake per bird could predict the DHA content of breast and thigh meat. DHA intake per bird was calculated by dividing the intake per pen by the number of birds present and then multiplying by the DHA content detected for each experimental diet.

## 3. Results

### 3.1. Ingredient and Diet Analysis

The AURA used in the study had a DHA content of 18%. The analytical composition of the experimental diets, including the predominant fatty acids detected in the feed are shown in Table 2.

### 3.2. Performance and Bird Health

The performance of the birds in terms of their weight gain, feed intake, and feed conversion ratio (FCR) is summarized in Table 3. During the first 21 days, positive improvements (*p* = 0.033) were observed between treatment groups in terms of weight gain, feed intake, and FCR. The group supplemented with 2.5% AURA had a significantly higher feed intake than the 0% and 5% groups, with a similar trend observed for weight gain with the 2.5% group gaining significantly more weight than the 0% group. From day 21–42 and for the study overall, no differences in productivity were observed among the treatment groups.

Mortality levels fell within the normal range for the facility, with 2.9%, 6.1%, 4.3%, and 5.4% mortality observed for the 0%, 0.5%, 2.5%, and 5% treatments, respectively. Results of the post mortems are available in Supplementary Table S1. No significant differences in the rate of mortality were observed between the groups (*p* = 0.148). Treatment had no effect on the blood hematology variables investigated (Table 4). Three blood serum biochemistry parameters were found to differ significantly among treatment groups: cholesterol levels were significantly lower (*p* = 0.02) in the 5% AURA group than in the control group; glutathione peroxidase was significantly higher (*p* = 0.02) in the 5% AURA group when compared to the control; and phosphate levels differed significantly (*p* = 0.04), however the Tukey test only detected a trend between the 2.5% and 0.5% (*p* = 0.055) and 2.5% and 0% (*p* = 0.055) AURA groups.

### 3.3. Meat Fatty Acid Content

**Figure 1:**
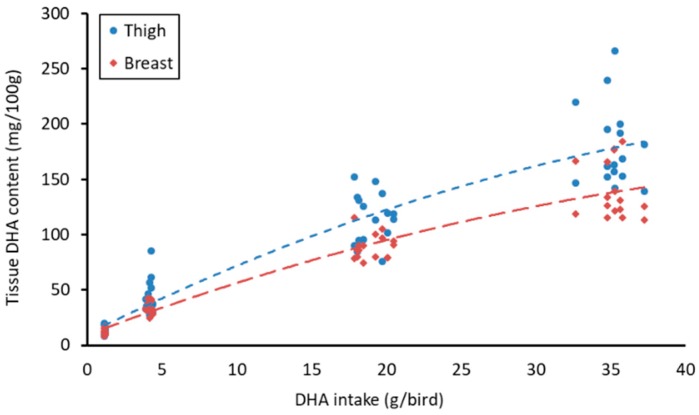
Scatterplot and regression lines for the estimated mean DHA intake (g/bird) against the DHA content (mg/100 g) detected in thigh and breast tissue samples taken after 42 days of supplementation with *Aurantiochytrium limacinum*. Thigh R^2^ = 0.8869, *p* < 0.001, Y = 10.27 + 6.701 X−0.05469 X^2^; breast R^2^ = 0.9214, *p* < 0.001, Y = 9.512 + 5.086 X−0.04049 X^2^. DHA intake per bird was estimated by dividing the intake per pen by the number of birds present and then multiplying by the DHA content detected for each experimental diet. The tissue analysis results for two birds per pen were then graphed against the corresponding estimated DHA intake for that pen.

The effects of supplementation with AURA on breast (m. *pectoralis major*) and thigh (m. *biceps femoris*) fatty acid content are summarized in Table 5 and Table 6, respectively. A quadratic increase in meat DHA content was observed with increasing DHA intake for both breast (R^2^ = 0.9577, *p* < 0.001) and thigh (R^2^ = 0.9639, *p* < 0.001) meat samples (Figure 1), with any increase in dietary inclusion of AURA corresponding to a significant increase in DHA content (Table 5 and Table 6). The content of EPA for both breast and thigh samples was significantly higher in the 2.5% and 5% treatments than the control and 0.5% treatments. Overall, the total n-3 FA content significantly increased with increasing AURA inclusion level (2.5% and 5%). Similar trends were observed for the thigh and breast samples with dose related increases observed for C20:4 n-3, EPA, and DHA, all contributing to the higher n-3 FA total. Only the breast tissue of the 5% treatment had significantly more n-3 DPA than the control and 0.5% groups, and while higher levels of n-3 DPA were observed in the thigh tissue of the 2.5% and 5% treatments, the difference among the treatments only approached significance (*p* = 0.069). The total n-6 FA did not differ significantly among the groups. However, a number of individual n-6 FA did significantly decrease, in a dose-dependent manner for both breast and thigh samples, with increasing AURA inclusion (i.e., C18:3 n-6, C20:3 n-6, C20:4 n-6, C22:4 n-6). C22:5 n-6 was the only n-6 FA to increase with 2.5% and 5% AURA supplementation in breast and thigh samples. As a result of these changes, the n-6/n-3 ratios significantly decreased with increasing AURA inclusion level for both breast and thigh tissues.

## 4. Discussion

This study was designed to assess the level of tolerance of broilers to life-long exposure to dietary AURA, at the intended use level (1X, i.e., 0.5%), and multiples of the intended use level, (5X and 10X, i.e., 2.5% and 5%, respectively), to establish safety and a limit of tolerance for the target animal [20]. With no differences in mortality observed between the groups, AURA was found to be well tolerated and safely consumed at up to ten times the intended use level. In addition, no differences in the blood hematology variables were observed between the groups. Yan et al. [22] reported significant increases in lymphocyte concentrations when supplementing the diets of broilers with the related microalgae *Schizochytrium* and suggested that algae extracts may stimulate an immune response. In the current study, the lymphocyte concentrations were numerically higher in the treatment groups than in the control, but these differences were not significant indicating that supplementation did not initiate an immune response in our case.

In terms of blood biochemical parameters, supplementation increased the level of glutathione peroxidase in the 5% AURA supplemented group, compared to the control. Glutathione peroxidase is an antioxidant enzyme which protects the organism from oxidative damage. It has been suggested that an increased polyunsaturated fatty acid (PUFA) intake may induce the production of antioxidant enzymes in broiler muscle [23,24]. In addition, the cholesterol concentration of the 5% group was significantly reduced when compared to the control group. Various diets with high PUFA concentrations have been shown to decrease serum total cholesterol in broilers, with the decrease attributed to a suppression of hepatic cholesterol production by the high PUFA levels present [25]. Productivity was not negatively impacted by supplementation. During the starter period of the study (D0–21) weight gain and feed intake increased for the 2.5% group compared to the control. No differences were observed among the groups during the grower period or overall indicating that productivity was not affected by AURA supplementation. This agrees with other authors who found supplementation with either fish oil or *Schizochytrium* had no effect on broiler productivity parameters [10,22]. With no negative impacts observed in terms of mortality, blood parameters or productivity, AURA was considered to be well tolerated by broilers.

In the European Union, food must contain at least 40 mg EPA + DHA per 100 g to make the nutritional claim that it is a source of n-3 fatty acids, while a food must contain at least 80 mg EPA + DHA to be considered high in omega-3 [26]. In the current study, at the intended use level (0.5%), breast meat had 38 mg/100 g of EPA + DHA falling just short of the 40 mg content required to be considered as a source of omega-3. The thigh meat, however, did meet this definition, with 47 mg/100 g EPA + DHA detected. Based on the regression analysis, DHA intake should be increased to 16 and 12 g of DHA/bird for breast and thigh, respectively, to be considered high in omega-3. As concluded from these results, dietary inclusion of approximately 2% would enrich breast meat to 80 mg DHA/100 g and thigh meat to 104 mg DHA/100 g. Meat enriched to these levels could contribute a significant proportion of the 250 mg of DHA that is considered as an adequate intake [27]. However, as the regression analysis is based on an estimated intake per bird, the actual increase required to reach this level of enrichment could differ. The highest levels of enrichment were observed with the highest levels of supplementation in which 140 and 180 mg DHA/100 g tissue were detected in the breast and thigh, respectively. However, in practice, supplementation at this level could not be used. In addition, supplementation at this level can result in a reduction in the consumer acceptability of broiler meat. Ribeiro et al. [28] reported that supplementation with *Schizochytrium* at a level of 7.4% of the diet negatively impacted consumer acceptability. When comparing the level of enrichment in the current tolerance study with that of Ribeiro et al. [28], similar levels of DHA were detected (139 vs. 120 and 179 vs. 310 mg/100 g in breast and thigh, respectively) which may suggest that the flavor of the meat from this study could also be negatively affected. At a lower inclusion level of 3.7%, the sensory qualities of enriched meat were not affected while meat DHA content was significantly increased [28]. Similar levels of DHA were detected in the meat of the 2.5% treatment in the current study and the 3.7% treatment in the Ribeiro et al. [28] experiment (89 vs. 70 and 115 vs. 150 mg/100 g in breast and thigh, respectively) indicating that the sensorial quality of the meat at this level and below may not be negatively affected. When comparing the use of *Schizochytrium* sp. and fish oil in terms of flavor quality, Mooney et al. [14] found supplementation with 5.5% algae and 2.1% menhaden oil significantly reduced flavor scores, however, supplementing 2.8% algae enriched the meat to the same degree as menhaden oil, while maintaining consumer acceptability.

Algae supplements are predominantly composed of DHA in comparison to fish oils which can have a significant amount of both EPA and DHA [14]. Despite the lack of EPA detected in the experimental diets, significant increases in breast and thigh were observed. This is in agreement with the findings of other authors who have demonstrated increased tissue EPA content when supplementing broilers with DHA-rich microalgae [14,28]. In the absence of supplementary EPA, these increases are likely due to the retro-conversion of DHA to EPA [29]. Significant increases in the total n-3 FA content were primarily due to the increased deposition of DHA, however significant increases of EPA and C20:4 n-3 also contributed to the total sum of n-3 FA. In contrast, no differences in the total n-6 content were observed despite the significant decreases for the majority of individual n-6 FA. The presence of C22:5 n-6 in the algae supplemented diets resulted in increasing concentrations of this fatty acid in the tissue samples and limited the general trend of decreasing n-6 concentration. However, the ratio of n-6 to n-3 FA was found to decrease significantly with increasing AURA supplementation. Intake of diets with a balanced n-6:n3 ratio of 1:1 is desirable, however in many parts of the world, the n-6 intake is much greater resulting in dietary intakes of >10:1 in many cases [30]. Reductions of this ratio ranging between 5:1 and 1:1, have been associated with improvements for a number of different health problems [30]. As such, supplementation of broilers with the DHA-rich AURA microalgae significantly improved this ratio, further increasing the nutritional value of the meat. The results of this study indicate that dietary supplementation with the microalgae AURA, does not negatively impact broiler health or productivity, and significantly improves the nutritional quality of the meat in terms of the omega-3 content and the n:6:n-3 ratio.

## Figures and Tables

**Table 1:** Ingredient and nutrient composition (%) of the basal experimental diets.

Diet Ingredients	Starter	Grower
Maize	64.042	66.902
Soya oil	1.637	2.501
DL-Methionine	0.267	0.225
L-Threonine	0.072	0.055
L-Lysine HCI	0.272	0.248
Limestone	0.528	0.427
Dicalcium phosphate	1.827	1.523
Sodium bicarbonate	0.341	0.344
Salt	0.150	0.150
Hipro soya	30.364	27.125
RNL vit/min ^1^	0.500	0.500
Diet nutrients		
Dry matter	87.8	87.864
Crude protein	20.25	18.854
Crude fat	4.47	5.360
Crude fiber	2.31	2.260
Ash	6	5.429
Starch (ewers)	41.2	43.034
Sugar	4.408	4.132
Calcium	0.9	0.780
Phosphorous P	0.667	0.598
Sodium	0.16	0.160
Potassium	0.868	0.804
Chloride	0.19	0.185
Lysine	1.268	1.159
Methionine	0.577	0.518
Methionine + cysteine	0.909	0.832
Threonine	0.826	0.756
Tryptophan	0.226	0.208
ME ^2^ broiler	2800	2900

^1^ Roslin Nutrition Vitamin/Mineral Premix supplies per kg feed: Vit A: 0.010 MIU; Vit D3: 0.005 MIU; Vit E: 50 mg; Vit K3: 3 mg; Vit B1: 2.0 mg; Vit B2: 7 mg; Vit B6: 5 mg; Vit B12: 15 mg; Folic acid: 1.0 mg; Biotin: 0.2 mg; Pantothenic acid: 15 mg; Niacinamide: 50 mg; Mo 0.5 mg; Mn: 100 mg; Zn: 80 mg; I: 1.0 mg; Fe: 80 mg; Cu: 10 mg; Se: 0.20 mg. ^2^ ME = Metabolizable energy.

**Table 2:** Analytical composition (%) concentration of selected fatty acids (mg fatty acid/g) of the starter and grower experimental diets supplemented with an unextracted *Aurantiochytrium limacinum* biomass at a rate of 0%, 0.5%, 2.5%, and 5%.

	Starter D0–21				Grower D22–42			
Nutrient Values	0.0%	0.5%	2.5%	5.0%	0.0%	0.5%	2.5%	5.0%
Dry matter	87.0	87.1	87.2	86.8	87.8	87.6	87.6	87.9
Crude fat (Oil A)	5.6	5.5	5.7	5.6	5.0	5.4	5.5	5.4
Crude protein	20.0	20.2	20.4	20.4	18.7	18.6	18.6	18.4
Ash	5.8	5.8	5.6	5.3	4.9	4.8	4.8	4.8
Fatty acid ^1^ content (mg/g)								
C14:0 Myristic acid	0.1	0.2	0.7	1.3	0.1	0.2	0.7	1.3
C16:0 Palmitic acid	6.8	7.9	13.7	20.7	7.2	8.4	14.8	20.9
C18:2 n-6 (LA)	23.0	22.7	22.9	23.0	27.9	27.5	28.2	25.9
C18:3 n-3 (ALA)	1.8	1.7	1.8	1.8	2.4	2.3	2.4	2.3
C20:5 n-3 (EPA)	0.0	0.0	0.0	0.1	0.0	0.0	0.0	0.1
C22:5 n-6 (DPA)	0.1	0.2	0.7	1.4	0.0	0.1	0.7	1.4
C22:6 n-3 (DHA)	0.3	0.9	3.7	7.1	0.0	0.6	3.7	6.9

^1^ Linoleic acid (LA); α-linolenic acid (ALA); eicosapentaenoic acid (EPA); n-6 docosapentaenoic acid (DPA); docosahexaenoic acid (DHA).

**Table 3:** The effect of dietary supplementation with an unextracted *Aurantiochytrium limacinum* biomass at a rate of 0%, 0.5%, 2.5%, and 5% on broiler performance parameters.

Trait	Age (day)	0%	0.5%	2.5%	5%	SE	*p*-Value
Weight gain (g)	0–21	843 ^b^	868 ^ab^	902 ^a^	870 ^ab^	21.3	0.033
Weight gain (g)	21–42	2234	2192	2250	2198	71.1	0.736
Weight gain (g)	0–42	3077	3060	3151	3069	83.3	0.562
Feed intake (g)	0–21	1107 ^b^	1159 ^ab^	1179 ^a^	1122 ^b^	24.8	0.010
Feed intake (g)	21–42	3904	3866	3961	3864	120.0	0.762
Feed intake (g)	0–42	5012	5025	5140	4986	133	0.553
^1^ FCR	0–21	1.32 ^ab^	1.34 ^a^	1.31 ^ab^	1.29 ^b^	0.01	0.002
FCR	21–42	1.75	1.76	1.76	1.76	0.02	0.579
FCR D0–42	0–42	1.63	1.64	1.63	1.62	0.001	0.086

^1^ Feed conversion ratio; ^a,b^ means within a row that do not share a superscript differ significantly.

**Table 4:** The effect of dietary supplementation with a DHA-rich unextracted microalgae, *Aurantiochytrium limacinum*, at a rate of 0%, 0.5%, 2.5%, and 5% on broiler blood biochemistry and hematology parameters.

Parameter	0%	0.5%	2.5%	5%	SEM ^1^	*p*-Value
Blood Biochemistry						
Alkaline phosphatase (IU/L)	13,663	13,298	11,919	13,877	1186	0.937
Aspartate amino transferase (IU/L)	618	630	616	415	31.5	0.067
Calcium (mmol/L)	2.34	2.33	2.39	2.31	0.03	0.861
Cholesterol (mmol/L)	3.69 ^b^	3.39 ^ab^	3.35 ^ab^	2.93 ^a^	0.08	0.019
Creatinine (µmol/L)	29.63	29.50	27.50	26.63	0.46	0.067
Glucose (mmol/L)	14.15	13.70	13.69	13.74	0.12	0.503
Glutathione peroxidase (u/mL RBC)	95.33 ^a^	112.16 ^ab^	112.20 ^ab^	119.96 ^b^	2.66	0.019
Lactose dehydrogenase (IU/L)	5161	4644	4153	2628	381	0.128
Magnesium (mmol/L)	0.963	0.963	0.988	0.988	0.01	0.607
Phosphate (mmol/L)	2.14 ^y^	2.14 ^y^	2.40 ^x^	2.19 ^xy^	0.03	0.035
Triglycerides (mmol/L)	0.726	0.726	0.818	0.731	0.05	0.868
Urea (mmol/L)	0.638	0.888	0.650	0.563	0.05	0.189
Uric acid (µmol/L)	357	296	380	362	22.08	0.571
*Blood hematology*						
Basophil (×10^3^/µL)	0.18	0.37	0.13	0.39	0.06	0.375
Eosinophil (×10^3^/µL)	0.16	0.44	0.60	0.60	0.08	0.191
Hemoglobin (g/L)	96.13	94.14	91.38	98.25	1.81	0.575
Heterophil (×10^3^/µL)	8.39	6.10	7.64	8.93	0.62	0.433
Lymphocyte (×10^3^/µL)	2.51	2.84	2.76	2.89	0.26	0.956
Mean corpuscular hemoglobin (g/L)	277	302	270	274	5.83	0.241
Mean corpuscular volume (fL)	181	164	174	181	4.12	0.440
Monocyte (×10^3^/µL)	0.20	0.40	0.60	0.40	0.07	0.301
Packed cell volume (l/L)	0.35	0.32	0.34	0.36	0.01	0.258
Red blood cell count (×10^6^/µL)	1.98	1.96	1.95	2.00	0.05	0.984
White blood cell count (×10^3^/µL)	11.48	10.16	11.81	13.19	0.70	0.526

^a,b^ Means with different letters in the same row differ significantly (*p* ≤ 0.05); ^x,y,z^ means with different letters in the same row differ (0.05 ≤ *p* ≤ 0.1).

**Table 5:** The effect of supplementation with a docosahexaenoic-acid rich unextracted *Aurantiochytrium limacinum* biomass (AURA) at a rate of 0%, 0.5%, 2.5%, and 5% of the diet on broiler on breast tissue fatty acid content.

Fatty Acid (mg/100 g)	0%	0.5%	2.5%	5%	SEM	*p*-Value
C14:0	7.12 ^b^	6.42 ^b^	8.76 ^b^	14.34 ^a^	2.18	<0.001
C16:0	365.61	300.06	283.62	337.15	66.1	0.469
C18:2 n-6 (LA)	401.10	319.93	265.28	295.18	75.0	0.187
C18:3 n-6 (GLA)	3.76 ^a^	2.38 ^ab^	1.54 ^b^	1.37 ^b^	0.75	0.001
C18:3 n-3 (ALA)	26.32	19.81	17.50	21.07	5.91	0.359
C18:4 n-3 (SDA)	0.93	0.48	0.39	0.77	0.30	0.125
C20:2 n-6	8.15	8.11	7.18	6.83	0.62	0.032
C20:3 n-6	12.20 ^a^	11.21 ^a^	8.73 ^b^	7.19 ^c^	0.64	<0.001
C20:4 n-6 (AA)	57.80 ^a^	51.32 ^b^	36.58 ^c^	32.45 ^c^	2.16	<0.001
C20:3 n-3	0.65 ^b^	1.05 ^a^	0.89 ^ab^	0.94 ^ab^	0.15	0.026
C20:4 n-3	0.14 ^b^	0.15 ^b^	0.31 ^b^	1.08 ^a^	0.19	<0.001
C20:5 n-3 (EPA)	3.28 ^c^	3.55 ^c^	6.59 ^b^	10.14 ^a^	0.71	<0.001
C22:4 n-6	13.80 ^a^	10.42 ^b^	4.16 ^c^	2.61 ^d^	0.50	<0.001
C22:5 n-6 (n-6 DPA)	3.11 ^c^	4.85 ^c^	9.25 ^b^	14.75 ^a^	0.99	<0.001
C22:5 n-3 (n-3 DPA)	9.02 ^b^	9.02 ^b^	10.19 ^ab^	10.52 ^a^	0.60	0.005
C22:6 n-3 (DHA)	12.20 ^d^	34.49 ^c^	89.06 ^b^	139.50 ^a^	6.03	<0.001
Total n-3 FA	52.54 ^c^	68.55 ^c^	124.92 ^b^	184.02 ^a^	11.7	<0.001
Total n-6 FA	499.92	408.21	332.72	360.39	78.5	0.078
n-6/n-3 ratio	9.33 ^a^	5.78 ^b^	2.57 ^c^	1.89 ^c^	0.37	<0.001

^a,b,c,d^ Means within a row that do not share a superscript differ significantly. The fatty acid abbreviations correspond to: Linoleic acid (LA); γ-linolenic acid (GLA); α-linolenic acid (ALA); Stearidonic acid (SDA); Arachidonic acid (AA); Eicosapentaenoic acid (EPA); n-6 Docosapentaenoic acid (n-6 DPA); n-3 Docosapentaenoic acid (n-3 DPA); Docosahexaenoic acid (DHA).

**Table 6:** The effect of supplementation with a docosahexaenoic-acid rich unextracted *Aurantiochytrium limacinum* biomass at a rate of 0%, 0.5%, 2.5%, and 5% of the diet on broiler on thigh tissue fatty acid content.

Fatty Acid (mg/100 g)	0%	0.5%	2.5%	5%	SEM	*p*-Value
C14:0	8.75 ^b^	11.94 ^b^	15.92 ^ab^	22.32 ^a^	3.45	<0.001
C16:0	442.28	512.1	465.95	489.95	100	0.861
C18:2 n-6 (LA)	504.1	575.7	465.0	443.1	115	0.552
C18:3 n-6 (GLA)	3.77 ^ab^	3.95 ^a^	2.43 ^ab^	1.94 ^b^	0.82	0.012
C18:3 n-3 (ALA)	32.40	38.07	32.87	32.75	9.15	0.868
C18:4 n-3 (SDA)	0.95	1.13	1.01	1.26	0.35	0.735
C20:2 n-6	8.55	9.43	8.63	8.24	1.04	0.579
C20:3 n-6	13.41 ^a^	14.05 ^a^	11.38 ^ab^	9.38 ^b^	1.31	<0.001
C20:4 n-6 (AA)	67.51 ^a^	65.53 ^a^	46.95 ^b^	42.67 ^b^	6.97	<0.001
C20:3 n-3	1.03	1.26	1.26	1.22	0.15	0.230
C20:4 n-3	0.28 ^c^	0.52 ^bc^	0.97 ^b^	1.76 ^a^	0.27	<0.001
C20:5 n-3 (EPA)	3.16 ^c^	4.71 ^c^	10.12 ^b^	15.66 ^a^	1.65	<0.001
C22:4 n-6	15.26 ^a^	12.12^b^	5.19 ^c^	3.50 ^c^	1.20	<0.001
C22:5 n-6 (n-6 DPA)	3.75 ^c^	6.30 ^c^	12.83 ^b^	20.16 ^a^	1.49	<0.001
C22:5 n-3 (n-3 DPA)	10.63	10.73	12.21	12.94	1.19	0.069
C22:6 n-3 (DHA)	14.26 ^d^	42.55^c^	114.77 ^b^	179.84 ^a^	9.77	<0.001
Total n-3 FA	62.69 ^c^	98.97 ^c^	173.21 ^b^	245.43 ^a^	20.8	<0.001
Total n-6 FA	616.39	687.08	552.41	529.02	126	0.463
n-6/n-3 ratio	9.74 ^a^	6.68 ^b^	3.07 ^c^	2.10 ^d^	0.12	<0.001

^a,b,c,d^ Means within a row that do not share a superscript differ significantly. The fatty acid abbreviations correspond to: Linoleic acid (LA); γ-linolenic acid (GLA); α-linolenic acid (ALA); Stearidonic acid (SDA); Arachidonic acid (AA); Eicosapentaenoic acid (EPA); n-6 Docosapentaenoic acid (n-6 DPA); n-3 Docosapentaenoic acid (n-3 DPA); Docosahexaenoic acid (DHA).

## Supplementary Materials

The following are available online at http://www.mdpi.com/2076-2615/8/10/180/s1, Table S1: Mortality and post-mortem results.
