# Supplementary material for: Tolerance of Broilers to Dietary Supplementation with High Levels of the DHA-Rich Microalga, Aurantiochytrium Limacinum: Effects on Health and Productivity
Source: Animals (Basel). 2018 Oct 16;8(10):180. doi: 10.3390/ani8100180 (PMC6211075; doi:10.3390/ani8100180)
Supplement: Supplementary file 1 [file animals-08-00180-s001.pdf]

Supplementary Table S1. Mortality and post-mortem results.

Treatment	Date	Weight (kg)	*Post mortem* outcome
0.0 %	13/03/2017	0.06	*N/A – too small*
	14/03/2017	0.12	*N/A – too small*
	23/03/2017	0.5	*Sudden Death Syndrome*
	26/03/2017	0.32	*N/A – off legs*
	29/03/2017	0.81	*N/A – off legs*
	09/04/2017	2.08	*Ascites*
	13/04/2017	1.74	*Ascites*
	13/04/2017	1.7	*Ascites*
0.5 %	14/03/2017	0.29	*N/A – off legs*
	18/03/2017	0.48	*Sudden Death Syndrome*
	20/03/2017	0.24	*N/A – too small*
	22/03/2017	0.98	*Sudden Death Syndrome*
	28/03/2017	1.18	*Sudden Death Syndrome*
	28/03/2017	0.56	*N/A – off legs*
	31/03/2017	1.6	*Enlarged Liver*
	04/04/2017	1.58	*Ascetic – heart failure*
	06/04/2017	1.84	*Ascites*
	07/04/2017	1.62	*Ascites*
	07/04/2017	1.44	*Cervical discolouration, dehydrated*
	09/04/2017	1.56	*Ascites*
	10/04/2017	2.28	*Ascites*
	10/04/2017	1.62	*N/A – off legs*
	11/04/2017	2.8	*Pericardial effusion, hepatomegaly*
	12/04/2017	2.26	*Ascites*
	12/04/2017	2.22	*Ascites*
2.5 %	14/03/2017	0.26	*Sudden Death Syndrome*
	20/03/2017	0.44	*N/A – off legs*
	22/03/2017	0.94	*Ascites*
	23/03/2017	0.32	*N/A – off legs*
	27/03/2017	1.52	*N/A – off legs*
	30/03/2017	1.42	*Ascites*
	03/04/2017	1.3	*N/A – off legs*
	07/04/2017	1.48	*Cervical discolouration*
	10/04/2017	1.91	*N/A – off legs*
	12/04/2017	2.28	*Ascites*
	12/04/2017	2.66	*Ascites*
	12/04/2017	2.48	*Ascites*
5 %	13/03/2017	0.3	*N/A – off legs*
	14/03/2017	0.3	*N/A – off legs*
	16/03/2017	0.22	*N/A - decomposed*
	19/03/2017	0.52	*Sudden Death Syndrome*
	20/03/2017	0.54	*N/A – off legs*
	24/03/2017	0.94	*Sudden Death Syndrome*
	27/03/2017	1.1	*N/A – off legs*
	27/03/2017	1.24	*Sudden Death Syndrome*
	28/03/2017	0.92	*Peritonitis*
	28/03/2017	0.54	*N/A – off legs*
	29/03/2017	0.51	*N/A – off legs*
	04/04/2017	0.88	*Sudden Death Syndrome*
	06/04/2017	1.3	*N/A – off legs*
	08/04/2017	1.2	*N/A – off legs*
	11/04/2017	2.11	*N/A – off legs*
